# Association of miRNA-145 with the occurrence and prognosis of hydrosalpinx-induced defective endometrial receptivity

**DOI:** 10.17305/bjbms.2020.4538

**Published:** 2021-02

**Authors:** Qingli Wang, Haiquan Ai, Xia Li, Haiqing Tian, Bingxue Ning, Meng Zhang, Xiaolin La

**Affiliations:** Department of Reproductive Medicine Center, The First Affiliated Hospital of XinJiang Medical University, Urumqi, XinJiang, China

**Keywords:** miRNA-145, hydrosalpinx, infertility, inflammation, endometrial cells

## Abstract

MiR-145 is reported to facilitate inflammation and is also associated with unsuccessful embryonic implantation. Whether miR-145 mediates inflammatory response underlying hydrosalpinx-induced defective endometrial receptivity (ER) remains unclear, and this study attempted to clarify this point. Endometrium samples were collected from hydrosalpinx patients (case, n = 243) and patients with tubal patency/obstruction (control, n = 187). The peripheral blood samples of cases and controls were collected to determine the genotypes of miR-145 SNPs. The value of miR-145 expression in the diagnosis and prognostic estimation of hydrosalpinx was assessed using ROC curve and regression analysis, respectively. Lipopolysaccharide (LPS) cell model was established with endometrial cells, and cells were transfected with miR-145 mimic, inhibitor, or negative control. MiR-145 and cytokine levels were quantified by quantitative reverse transcription PCR or western blot. MiR-145 expression was significantly higher in hydrosalpinx compared to control group, and high miR-145 expression was significantly associated with moderate/severe tube lesion, high pulsatility index (>1.06), and high resistance index (>0.61) in hydrosalpinx patients. ROC curve analysis indicated that monitoring miR-145 expression may be useful for the diagnosis of hydrosalpinx (AUC = 0.704). A alleles of rs41291957 (G>A) and rs353292 (G>A) were significantly associated with an increased risk of hydrosalpinx compared to G allele (*p* < 0.05), yet the mutant allele of rs353291 (A>G) and rs4705343 (T>C) significantly reduced susceptibility to hydrosalpinx compared to the wild type allele. Treatments with miR-145 mimic and LPS in endometrial cells significantly increased the levels of transforming growth factor-b1, tumor necrosis factor -a, interleukin (IL)-6, and IL-8 compared to negative control, while treatment with miR-145 inhibitor decreased the cytokine levels. In conclusion, abnormally expressed miR-145 may be involved in hydrosalpinx-induced ER defects by regulating the inflammatory response.

## INTRODUCTION

Hydrosalpinx, clinically embodied as a blockage in the distal end of the fallopian tube, triggers expansion of tubal wall and accumulation of fluid. Hydrosalpinx is a major contributor to defective endometrial receptivity (ER) [1], which increases the likelihood of spontaneous abortion and heterotopic pregnancy among females. There were several explanations for this causality, for instance, hydrosalpinx-induced endometrial cavity fluid could induce thinning of the endometrium [2-5], which impaired ER and hindered successful embryonic implantation [6]. Simultaneously, the hydrops scoured the uterine cavity and thereby blocked the adhesion of the embryo to the endometrium [7]. Besides, hydrosalpinx also led to abnormal expression of endometrial adhesion molecules (e.g., HOXA10) and inflammation biomarkers (e.g., interleukin [IL]-2), which altogether disturbed embryonic implantation [8,9]. Elucidating the pathogenesis of hydrosalpinx, such as inflammation disorder, is critical to prevent defective ER, which is a prerequisite for successful embryonic implantation.

There has been an increasing interest in microRNAs (miRNAs) associated with embryonic implantation and defective ER [10-12]. Specifically, miR-135a and miR-135b could trigger defective ER by reducing HOXA10 expression in endometrium [11]. Besides, miR-145 was found to hamper embryonic implantation by lowering insulin-like growth factor 1 receptor (IGF-1R) expression [12]. It is noteworthy that miR-145 was also inflammation-related [13]; for instance, suppressing miR-145 level could downregulate the expression of inflammatory factors (e.g., IL-5 and IL-13) [14]. MiR-145 was a regulator of Runx3, which affected the balance of Th1- and Th2-cytokines significantly [15]. Despite the association of miR-145 with ER and inflammation response, hardly any study directly elaborated the involvement of miR-145 in modifying hydrosalpinx-caused ER defect.

As revealed by the World Health Organization, approximately 15% of infertility cases were attributable to genetic variation, such as chromosome aberrations and genetic mutations [16]. Genetic mutations can affect expression and function of genes [17], eventually causing onset of diseases. For instance, rs4705342, a single nucleotide polymorphism (SNP) located in the promoter of miR-145 gene, was associated with differential expression of miR-145 [18], thereby elevating the risk of miR-145-mediated diseases. However, whether miR-145 SNPs are associated with defective ER remains unclear.

This study aimed at confirming the association of miR-145 SNPs with hydrosalpinx-induced defective ER. *In vitro* experiments were also performed to investigate the effect of miR-145 on inflammation, which could explain hydrosalpinx-induced infertility.

## MATERIALS AND METHODS

### General information on clinical samples

Endometrial samples were collected from hydrosalpinx patients (case, n = 243) and those with fallopian tube patency/obstruction (control, n = 187). All subjects were diagnosed by hysterosalpingography, and they received treatments at The First Affiliated Hospital of XinJiang Medical University from August 2017 to October 2018. The participants under following conditions were excluded from the hydrosalpinx group: 1) they were complicated by other infertility-related disorders, such as a drastic decline of ovarian function and follicle-stimulating hormone (FSH) of >18 U/L; 2) they performed ovarian/fallopian tube-relevant surgeries before; 3) they have experienced treatments relevant to endometriosis, intrauterine adhesions, endometrial polyps, or uterine malformations; and 4) their maldeveloped uterine volume and endometrium resulted from endocrine disorders. All participants signed informed consents, and the study was approved by The First Affiliated Hospital of XinJiang Medical University and its ethics committee.

### Inspection of patients

Colpo-ultrasonography was performed using a LOGIQ E9 color ultrasound scanner (GE Healthcare, USA) at the probe frequency of 5.0–9.0 MHz. From the 2^nd^ to 5^th^ days of the participant’s menstrual period, several hemodynamic parameters were recorded, including peak systolic velocity (PSV), end-diastolic velocity (EDV), pulsatility index (PI), and resistance index (RI). On their 10^th^ day, B-ultrasound was employed to determine the subject’s ovulation day. Meanwhile, bilateral endometrial thickness, uterine artery PI (UAPI), uterine artery RI (UARI), and endometrial blood flow of the subjects were also recorded. The endometrial blood perfusion was classified as: 1) type A in which blood flew through and beneath the intima, 2) type B in which blood merely flew under the endometrium, and 3) type C in which blood was unobservable in and beneath the endometrium [19,20]. By contrast, there were no specific time requirements for amenorrhea patients. Furthermore, disorders in the intraoperative pelvic and fallopian tube were assessed in accordance with standards stipulated by the American Society for Reproductive Medicine [21], and patients with bilateral hydrosalpinx were graded based on their side with severe lesions.

### Genotyping of miR-143/145-associated polymorphisms

Genomic DNAs were extracted from peripheral blood of hydrosalpinx patients and those carrying fallopian tube patency/obstruction, according to the instruction of Blood Genome Extraction Kit (TIANGEN, Germany). Polymerase chain reaction (PCR) was carried out under following conditions: 1) 95°C for 5 min, 2) 35 cycles of 95°C for 40 s, 60°C for 40 s, and 72°C for 60 s, and 3) 72°C for 5 min. PCR reaction system (25 ml) was composed of DNA template (1 ml), upstream primer (1 ml), downstream primer (1 ml), 2 × PCR TaqMix (12.5 ml), and ddH_2_O (9.5 ml). The primers for SNPs (Table S1) were synthesized by Sangon Biotech (Shanghai, China). After digestion at 37°C overnight, genotypes of the SNPs were identified utilizing 2.5% agarose gel electrophoresis and GoldViewI nucleic acid staining.

### Separation of endometrial cells

After being washed by Hanks’ solution for 3 times, the endometrial tissues were cut into pieces sized as 1–2 mm^3^ and then digested by 0.25% type-I collagenase (20 ml, Sigma, USA) for 2 h. Thereafter, the resultant mixture was centrifuged at 5000 r/min for 3 min, so as to collect supernatants. Furthermore, the precipitates were rinsed by Hank’s solution, and the supernatants were removed. Then, the precipitate was inoculated into culture bottles at a density of 4 × 10^2^ glands/cm^2^, and the supernatants were centrifuged at 1200 r/min for 10 min. Subsequently, cell suspension was adjusted to a density of 5 × 10^4^ cells/cm^2^. The cells were cultured in 5% CO_2_ at 37°C, and the culture solution was changed every 3 days. Finally, the cells were passaged until they spread over the wall.

### Culture of endometrial cells

After being dissociated by 0.25% trypsin for 10 min, Dulbecco’s Modified Eagle Medium/Nutrient Mixture F-12 (DMEM/F-12) that contained 15% fetal bovine serum (FBS) was dropped into cells to terminate digestion. The proportion of viable cells was confirmed to be >90% with the aid of trypan blue staining, and the density of the cells was then adjusted to 1 × 10^5^/ml. Afterward, the passaged cells were cultured in 5% CO_2_ at 37°C.

### Establishment of lipopolysaccharide (LPS) cell models

Endometrial cells at the logarithmic growth phase were treated by 0 ng/ml, 10 ng/ml, 50 ng/ml, 100 ng/ml, and 1000 ng/ml LPS, respectively. After 48-h cell incubation for 6 h, 12 h, 24 h and 48 h, 5 mg/ml MTT was supplemented to incubate the cells for another 4 h. Subsequently, the mixture was shaken for 5 min after addition of 100 μl dimethyl sulfoxide (DMSO). Finally, the absorbance (A) values of the cells were measured on the microplate reader at the wavelength of 570 nm, and the optimum concentration of LPS was determined when cell-inhibition rate (IR) became ≤10%. The IR was calculated according to the formula:.


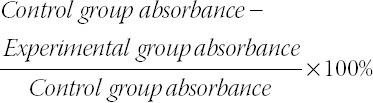


### Cell transfection

The endometrial cells at the exponential growth phase were inoculated into culture dishes at a density of 2.0 × 10^4^ per well, after being digested by 0.25% trypsin. When the endometrial cells grew to 80% confluence, miR-145 mimic (5’-GUCCAGUUUUCCCAGGAAUCCCU-3’) (GenePharma, China), miR-145 inhibitor (5’-AGGGAUUC CUGGGAAAACUGGAC-3’) (GenePharma, China), and miR-NC (5’-CAGUACUUUUGUGUAGUACAA-3’) (GenePharma, China) were transfected into the cells, according to the instruction of Lipofectamine 2000 Kit (Invitrogen, USA).

### Reverse transcription PCR (RT-PCR)

Total RNA was extracted from endometrial tissues and cell lines using TRIzol reagent (Invitrogen, USA) followed by reverse transcription (Invitrogen, USA) to synthesize cDNAs. Then, PCR was conducted with real-time PCR Master Mix Kit (TOYOBO, Japan), and primers of the genes were enlisted in Table S1. The miR-145 level and mRNA levels of transforming growth factor beta 1 (TGF-β1), tumor necrosis factor alpha (TNF-α), IL-6, and IL-8 were quantitated using the 2^−ΔΔCt^ method [22]. U6 was set as the internal reference for miR-145, while b-actin was utilized as the internal reference for cytokines.

### Western blotting

Proteins were isolated from endometrial tissues and cells by supplementation of 200 μl RIPA lysis buffer, which was made up of 50 mmol/l Tris-HCl (pH 7.5), 150 mmol/L NaCl, 1% NP-40, 0.5% sodium deoxycholate, and 0.1% sodium dodecyl sulfate (SDS). After centrifuging the lysates at a speed of 12 000 r/min for 10 min, the concentration of total protein was measured with BCA Kit (Pierce, USA). Then, 40 mg protein of each sample was separated by SDS-polyacrylamide gel electrophoresis (SDS-PAGE), and the products were then transferred onto polyvinylidene fluoride (PVDF) membrane. After blockage with 10% skimmed milk for 2 h, the samples were incubated by primary antibodies (Rabbit-Anti-Human, Abcam, USA) against TGF-β1 (1:1000, ab179695), TNF-α (1:500, ab6671), IL-6 (1:1000, ab6672), and IL-8 (1:10, ab7747) at 4°C. On the 2^nd^ day, after washing the membrane with Tris-Buffered Saline and Tween 20 (TBST) for 3 times, proteins were incubated by horseradish peroxidase (HRP)-labeled secondary antibody (goat anti-rabbit, 1:2000, ab6721, Abcam, USA) for 1 h. After development through chemiluminescence, gray values of the proteins were assessed using Image J software, and relative expressions of the proteins were quantitated with glyceraldehyde 3-phosphate dehydrogenase (GAPDH) as the internal reference.

### Statistical analysis

All data were statistically analyzed using SPSS Statistics for Windows, Version 17.0. (SPSS Inc., Chicago, USA). Measurement data, expressed as mean ± standard deviation (SD), were compared by Student’s *t*-test or one-way analysis of variance (ANOVA), and categorical data were compared utilizing *χ*^2^ test. Besides, correlation was assessed by Pearson correlation analysis, and receiver operating characteristic (ROC) curves were used to evaluate the diagnostic value of miR-145 for hydrosalpinx. Univariate and multivariate regression analyses were applied to explore the predictors of tube lesion severity in hydrosalpinx patients. A value of *p* < 0.05 was considered statistically significant.

## RESULTS

### Baseline characteristics of hydrosalpinx patients

**FIGURE 1:**
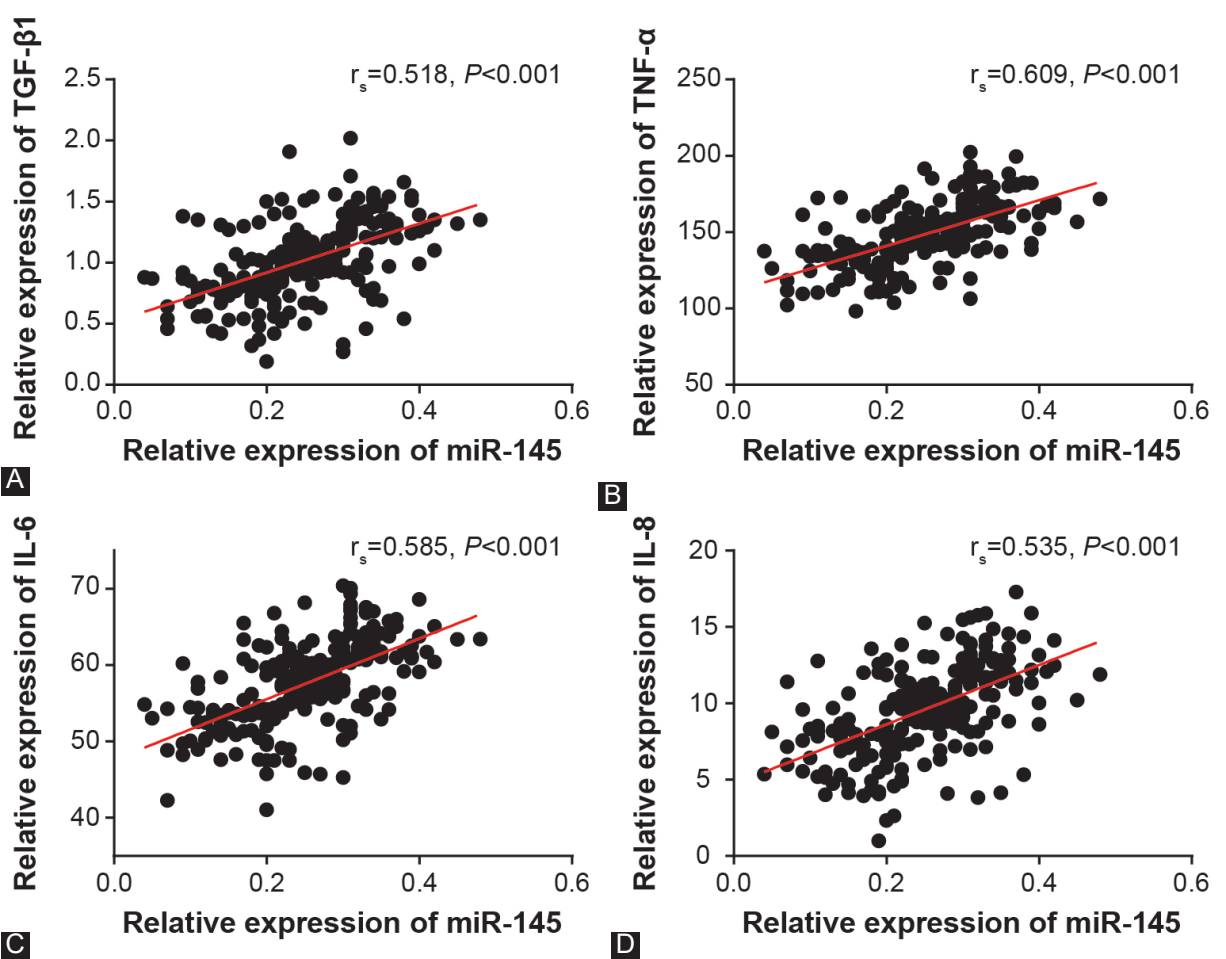
Correlations between miR-145 expression and transforming growth factor (TGF)-β1 (A), tumor necrosis factor (TNF)-α (B), interleukin (IL)-6 (C), and IL-8 (D) levels detected among the included hydrosalpinx patients.

Higher expression of miR-145 was detectable among hydrosalpinx patients than among the control population, and the hydrosalpinx group showed higher levels of TGF-β1, TNF-α, IL-6, and IL-8 than the control group (all *p* < 0.05; Table 1). The hydrosalpinx patients were associated with a larger proportion of type C endometrium, thinner intima, larger PI, and larger RI than control group (all *p* < 0.05; Table 1). Moreover, miR-145 expression showed positive correlations with serum levels of TGF-β1 (r_s_ = 0.518, *p* < 0.001), TNF-α (r_s_ = 0.609, *p* < 0.001), IL-6 (r_s_ = 0.585, *p* < 0.001), and IL-8 (r_s_ = 0.535, *p* < 0.001) among the hydrosalpinx patients (Figure 1).

**TABLE 1:**
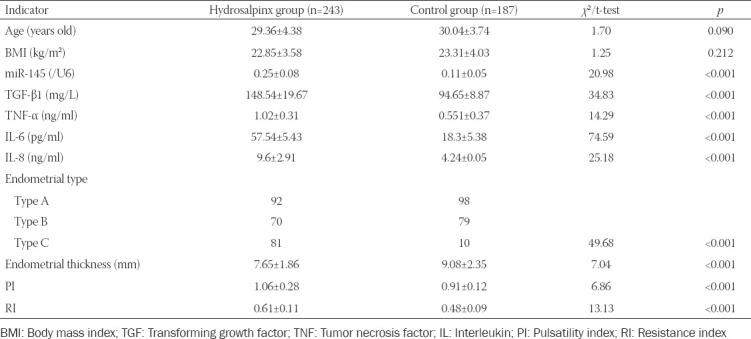
Comparison of clinical indicators between hydrosalpinx population and healthy controls

### Association of miR-145 expression with clinical features of hydrosalpinx patients

Utilizing the median value of miR-145 expression as the threshold, 243 hydrosalpinx cases were divided into highly-expressed miR-145 group (n = 164) and lowly-expressed miR-145 group (n = 79). The results of univariate regression analysis suggested that high miR-145 expression was associated with moderate/severe tube lesion, PI of >1.06, and RI of >0.61 of hydrosalpinx patients (*p* < 0.05; Table 2). The multivariate regression analysis further indicated that highly-expressed miR-145, moderate/severe tube lesion, and PI of >1.06 were independent predictors of hydrosalpinx-induced infertility (all *p* < 0.05; Table 3).

**TABLE 2:**
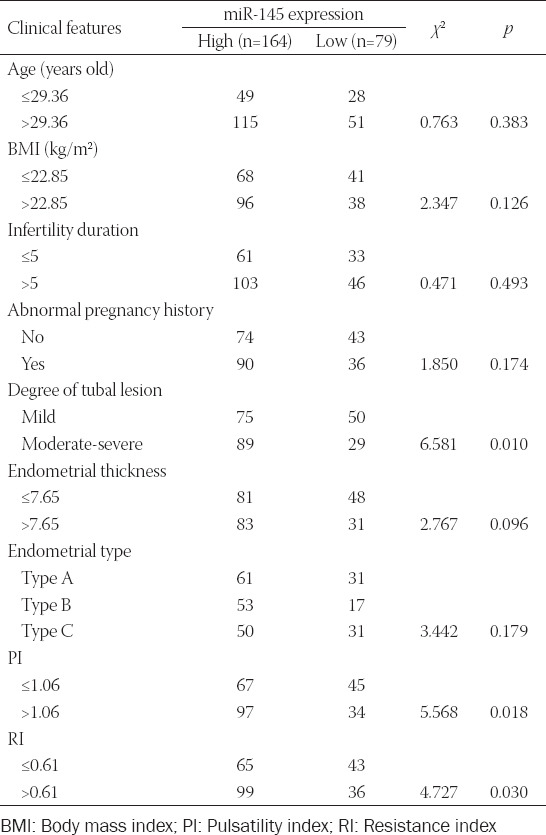
Association of miR-145 expression with clinical features of hydrosalpinx patients

**TABLE 3:**
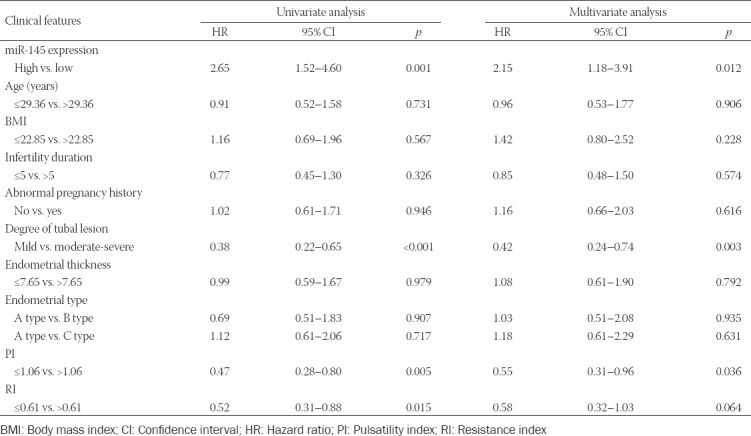
Association of clinical features and poor prognosis of hydrosalpinx patients

### Diagnostic performance of miR-145 for hydrosalpinx

**FIGURE 2:**
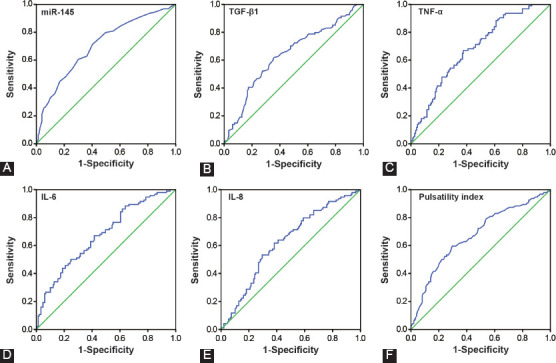
Diagnostic ability of miR-145 (A), transforming growth factor (TGF)-β1 (B), tumor necrosis factor (TNF)-α (C), interleukin (IL)-6 (D), IL-8 (E), and pulsatility index (F) in differentiating hydrosalpinx patients from healthy controls.

**FIGURE S1:**
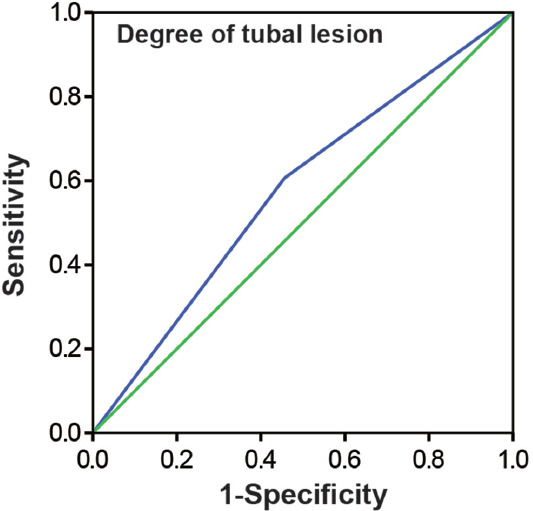
Diagnostic ability of tubal lesion degree in differentiating hydrosalpinx patients from healthy controls.

The serum level of miR-145 was capable of differentiating hydrosalpinx patients from those without hydrosalpinx (area under the curve [AUC] = 0.704) (Table 4, Figure 2A). TGF-β1 (AUC = 0.639), TNF-α (AUC = 0.681), IL-6 (AUC = 0.673), and IL-8 (AUC = 0.625) also demonstrated high diagnostic value for hydrosalpinx (Figure 2B-E). PI [AUC = 0.672] (Figure 2F) and tubal lesion degree [AUC = 0.575] (Figure S1) were also reliable measures for the diagnosis of hydrosalpinx.

**TABLE 4:**
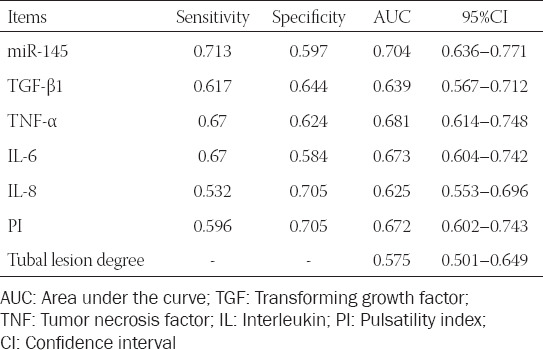
Diagnostic efficacy of miR-145 and clinical indicators in differentiating hydrosalpinx patients

### Association of SNPs and haplotypes of miR-145 with hydrosalpinx

Allele A of rs41291957 (G>A) and rs353292 (G>A) was found to increase the probability of hydrosalpinx onset, as compared with allele G (*p* < 0.05; Table 5). Conversely, rs353291 (A>G) and rs4705343 (T>C) decreased hydrosalpinx risk under their allelic models (*p* < 0.05). With respect to the dominant model, rs353291 (GG+AG/AA) and rs4705343 (CC+TC/TT) both reduced the risk of hydrosalpinx (*p* < 0.05), whereas rs41291957 (AA+GA/GG) raised susceptibility to hydrosalpinx (*p* < 0.05). When recessive models of the SNPs were considered, rs41291957 (AA/GG+GA) and rs353292 (AA/GG+GA) were associated with increased risk of hydrosalpinx (*p* < 0.05), while rs353291 (GG/AA+AG) significantly decreased the likelihood of hydrosalpinx onset (*p* < 0.05). In addition, the haplotype AAAT was associated with increased susceptibility to hydrosalpinx, while the haplotype GGAT acted as a protector against hydrosalpinx risk in comparison to other haplotypes (*p* < 0.05; Table 6).

**TABLE 5:**
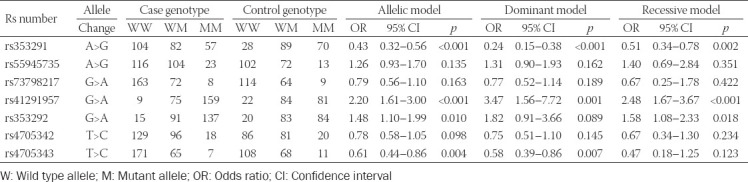
Association of genetic polymorphisms of miR-145 with onset of hydrosalpinx

**TABLE 6:**
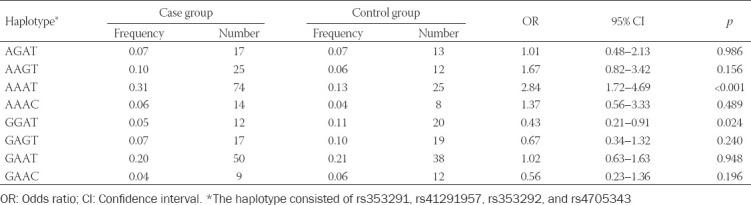
Association of haplotypes of miR-145 with onset of hydrosalpinx

### Correlation of SNPs and haplotypes of miR-145 with hydrosalpinx-related infertility

The possibility of pregnancy among hydrosalpinx patients was significantly reduced when they carried allele A of rs353292 (G>A), under allelic (A/G), dominant (AA+GA/GG), and recessive (AA/GG+GA) models (all *p* < 0.05; Table 7). Similarly, hydrosalpinx patients carrying allele A of rs41291957 (G>A) were more likely to suffer from infertility, when allelic model (A/G) and dominant model (AA+GA/GG) were considered (*p* < 0.05). By contrast, the incidence of non-pregnancy dropped significantly as they carried allele C of rs4705343 (T>C) (*p* < 0.05). Moreover, the haplotype GGC decreased the possibility of infertility among hydrosalpinx patients, when compared with other haplotypes (*p* < 0.05; Table 8). Concerning the results of stratified analyses, allele A of rs41291957 (G>A) or rs353292 (G>A) was suggestive of moderate/severe tubal lesion among hydrosalpinx patients (*p* < 0.05; Table S2), and allele A of rs41291957 (G>A) aggravated infertility risk among hydrosalpinx patients who were characterized by high PI (>1.06) or RI (>0.61) (*p* < 0.05; Table S3).

**TABLE 7:**
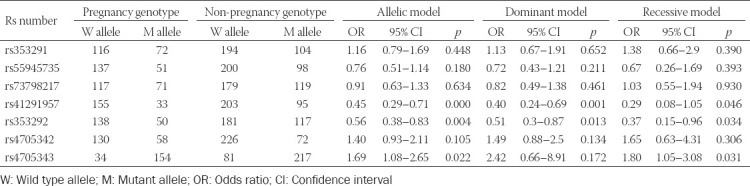
Association of genetic polymorphisms of miR-145 with prognosis of hydrosalpinx

**TABLE 8:**
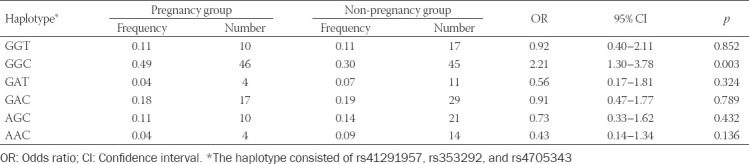
Association of haplotypes of miR-145 with prognosis of hydrosalpinx

### Effect of miR-145 on production of inflammatory factors by endometrial cells

**FIGURE 3:**
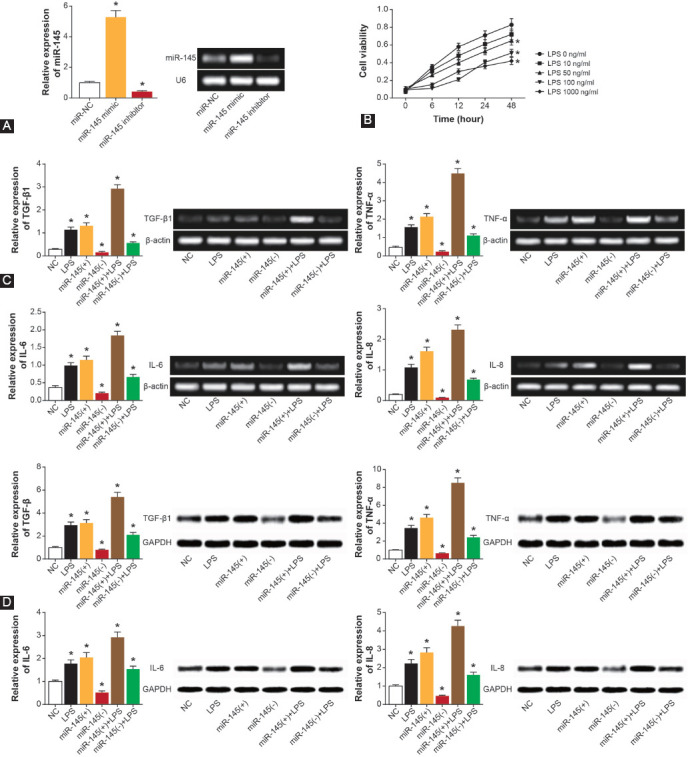
Effect of miR-145 on endometrial cell release of inflammatory cytokines. (A) Expression of miR-145 was identified after transfecting miR-145 mimic or miR-145 inhibitor into endometrial cells. *p < 0.05 when compared with miR-NC. (B) The viability of endometrial cells was determined after LPS treatments at 10–1000 ng/ml concentrations for 1–4 days. *p < 0.05 when compared with 0 ng/ml LPS treatment. (C) The mRNA levels of TGF-β1, TNF-α, IL-6, and IL-8 were obtained from endometrial cells treated with NC, LPS, miR-145 mimic, miR-145 inhibitor, miR-145 mimic+LPS, or miR-145 inhibitor+LPS. *p < 0.05 when compared with NC group. (D) The protein levels of TGF-β1, TNF-α, IL-6, and IL-8 were evaluated among endometrial cells of NC, LPS, miR-145 mimic, miR-145 inhibitor, miR-145 mimic+LPS, and miR-145 inhibitor+LPS groups. *p < 0.05 when compared with NC group. NC: Negative control; LPS: Lipopolysaccharide; TGF: Transforming growth factor; TNF: Tumor necrosis factor; IL: Interleukin.

The miR-145 expression within endometrial cells was increased considerably by miR-145 mimic (*p* < 0.05), and miR-145 expression in the miR-145 inhibitor group dropped significantly in comparison to NC group (*p* < 0.05; Figure 3A). Furthermore, the viability of endometrial cells increased when they were treated with 100 ng/ml LPS for 24 h (Figure 3B). In addition, LPS treatment significantly boosted the production of TGF-β1, TNF-α, IL-6, and IL-8 in endometrial cells (*p* < 0.05; Figure 3C and D), yet miR-145 mimic elevated the protein and mRNA levels of the inflammatory cytokines more significantly than LPS treatment. Conversely, miR-145 inhibitor resulted in a drastic reduction of TGF-β1, TNF-α, IL-6, and IL-8 levels in the endometrial cells (*p* < 0.05). Co-treatment of miR-145 mimic and LPS led to higher production of inflammatory cytokines than treatment of LPS or miR-145 mimic alone (*p* < 0.05). However, mRNA and protein levels of TGF-β1, TNF-α, IL-6, and IL-8 were lower in miR-145 inhibitor+LPS group than in LPS group (*p* < 0.05).

## DISCUSSION

Hydrosalpinx, mainly caused by the infection of the pelvic cavity or endometriosis, had considerable effects on embryonic transfer of females and even made them infertile [23]. A meta-analysis demonstrated that hydrosalpinx patients were more likely to fail in embryonic implantation than women without hydrosalpinx [24,25]. Moreover, a study showed that inflammatory cytokines and toxins in the uterine cavity were responsible for damaging the embryo of hydrosalpinx patients, and the expression of endometrial leukemia inhibitory factor (LIF), integrin b3, and mucoprotein 1 (MUC) also achieved a decline in hydrosalpinx patients [26]. Hydrosalpinx not only contributed to endometrial thinning and rapid movement of the myometrium, but it also changed the direction of endometrial movement, which altogether hampered successful embryonic implantation. Additionally, biomarkers indicative of vascularization were suppressed in hydrosalpinx patients, which ultimately led to difficulty in blastocyst implantation [27]. Among the etiologies, abnormal inflammation, a pivotal cause of infertility [28], was the focus of this study.

Accumulating evidence has emphasized the significance of miRNAs in triggering poor ER and infertility. For instance, miR-96 and miR-30b levels were obviously upregulated in embryonic implantation sites as compared with non-implantation sites [29], and defective ER was also inducible by high let-7a/7b expression [30]. In addition, miR-135a/b was reported to regulate expression of *HOXA10* [11], a gene involved in embryonic implantation and endometrial decidualization [31]. MiR-21 was also implicated in the etiology of defective ER, as it was positively regulated by LIF [32]. LIF is a multifunctional glycoprotein produced by endometrial cells [33-35], and one of its roles is promoting follicular growth and embryo implantation [36]. In addition, miRNA-145 was also documented to impair embryonic adhesion by directly reducing the expression of IGF-1R, which affected embryo implantation by interaction with IGF-1 [37,38] in endometrial epithelial cells [12]. IGF-1 facilitated embryo-to-blastula development and, conversely, depressing IGF-1 expression prevented the formation of blastula [39,40]. All these findings suggested that the interaction of IGF-1 with IGF-1R contributes to pre-implantation embryonic development, indicating the association of miR-145 with embryonic implantation. In this study, we confirmed that high miR-145 expression was associated with hydrosalpinx-induced infertility (Table 2 and 3, Figure 2). Moreover, the determination of miR-145-relevant genotypes (e.g., rs41291957, rs353292, rs353291, and rs4705343) may also help to predict infertility among patients with hydrosalpinx (Tables 5-8). However, whether miR-145 SNPs affect miR-145 expression in hydrosalpinx has been unclear, demanding more research.

Among the recruited hydrosalpinx patients, miR-145 level was positively correlated with the expression of TGF-β1, TNF-α, IL-6, and IL-8 (Figure 1). MiR-145 also facilitated the secretion of TNF-α, TGF-β1, IL-6, and IL-8 by endometrial cells (Figure 3), which indicated that miR-145 might be involved in hydrosalpinx etiology by enhancing the inflammation response. This was consistent with previous findings, which suggested that miR-145 could guide Th2-associated inflammation in myeloid cells by modulating the TGF-β pathway [41]. However, the effect of miR-145 on the viability of endometrial cells was not proved in this study, though former studies have shown that miR-145 refrained the viability of cancer cells [42].

## CONCLUSION

MiR-145 was associated with hydrosalpinx-induced infertility through regulating the inflammation response of endometrial cells. However, there are several pitfalls in the experimental design of this study. First, we failed to investigate the downstream genes of miR-145 underlying pathogenesis of hydrosalpinx-caused infertility. Second, animal models were not used to verify the role of miR-145 in modulating inflammatory disorder inherent in infertility etiology. More in-depth experiments should be conducted to address these problems and confirm our results.

## Figures and Tables

**TABLE S1:**
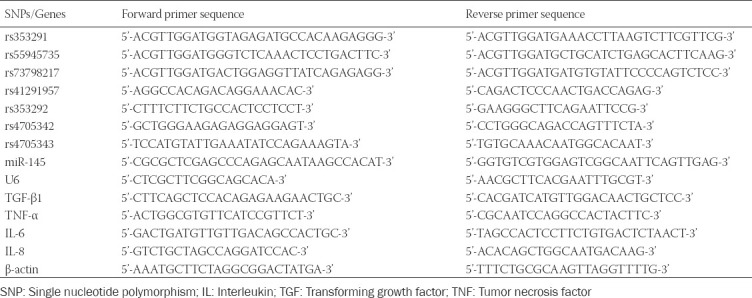
Primers for genotyping miR-145-related polymorphisms and polymerase chain reaction of miR-145 and relevant cytokines

**TABLE S2:**
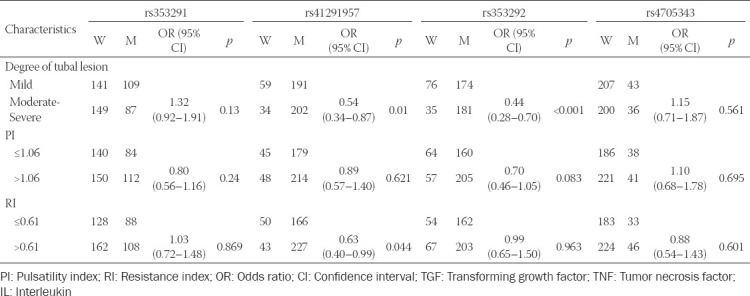
Stratified analyses of association of significant polymorphisms in miR-145 with clinical features of hydrosalpinx patients

**TABLE S3:**
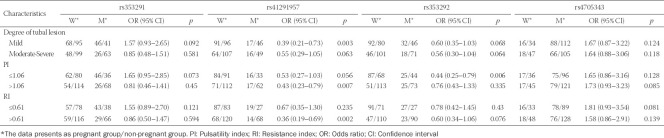
Stratified analyses of association of significant polymorphisms in miR-145 with pregnancy in hydrosalpinx patients
